# Remodeling the gut microbiota: mechanistic insights into probiotic-mediated alleviation of weaning stress in piglets

**DOI:** 10.3389/fimmu.2026.1964247

**Published:** 2026-09-28

**Authors:** Wenxiao Zhuo, Ziding Yu, Kuanhui Liu, Wenwen Zhang, Zhenxing Ma, Zejun Xu, Julong Wang, Ting Li, Guangshuang Zhu, Benzhong Wang, Ran Xiong, Chao Li, Haiyan Zhang, Weiwei Wang

**Affiliations:** 1 Wuhu Vocational Technical University, Wuhu, China; 2 Detection of Food-borne Pathogenic Microorganisms Engineering Research Center of Wuhu, Wuhu, China; 3 Anhui Science and Technology University, Chuzhou, China; 4 Anhui Province Key Laboratory of Animal Nutritional Regulation and Health, Chuzhou, China

**Keywords:** gut microbiota, intestinal homeostasis, microbiota remodeling, piglets, probiotics, short-chain fatty acids, weaning stress

## Abstract

Early weaning, characterized by the replacement of maternal milk with solid diets, is widely recognized as one of the most stressful developmental events affecting piglets in intensive production systems. During this stage, the digestive, immune, and metabolic systems remain immature, rendering piglets highly susceptible to environmental and nutritional stressors as well as opportunistic pathogens. These challenges frequently disrupt intestinal microbial homeostasis, impair gut barrier integrity, and trigger immune and metabolic disturbances, ultimately leading to post-weaning diarrhea, intestinal inflammation, growth retardation, and reduced production performance. For decades, dietary antibiotics have been extensively used to mitigate weaning-associated diseases. However, the emergence and dissemination of antimicrobial resistance, together with concerns regarding antibiotic residues and microbiota disruption, accelerated the search for safe, effective, and sustainable alternatives to maintain intestinal health in antibiotic-free swine production. As one of the most promising microbiota-targeted strategies, probiotics have demonstrated considerable potential for alleviating weaning stress by restoring the structure and function of the gut microbial ecosystem. This review systematically summarizes weaning-induced alterations in gut microbial composition, ecological succession, and microbial metabolic dysfunction, and discusses how these changes contribute to intestinal dysfunction in piglets. Emphasis is placed on the mechanisms by which probiotics remodel the intestinal microbiota, promote the establishment of health-associated microbial communities, and restore key microbial metabolic pathways, including short-chain fatty acid (SCFA), secondary bile acid, and tryptophan metabolism. Although probiotics may interact directly with host tissues, their effects are primarily mediated through restoration of the gut microbial ecosystem and re-establishing microbiota-derived metabolic signaling, thereby coordinating intestinal barrier repair, immune homeostasis, and redox balance while alleviating post-weaning diarrhea and growth impairment. Probiotic-mediated restoration of gut microbial structure and metabolic function represents the central mechanism underlying protection against weaning stress and can be conceptualized as a sequential process involving microbial ecosystem reconstruction, metabolic recovery, and host functional restoration. These insights provide a mechanistic framework for developing precision microbiome-based interventions and sustainable antibiotic-free strategies to improve intestinal health and production efficiency in modern swine production systems.

## Graphical Abstract

**Figure:**
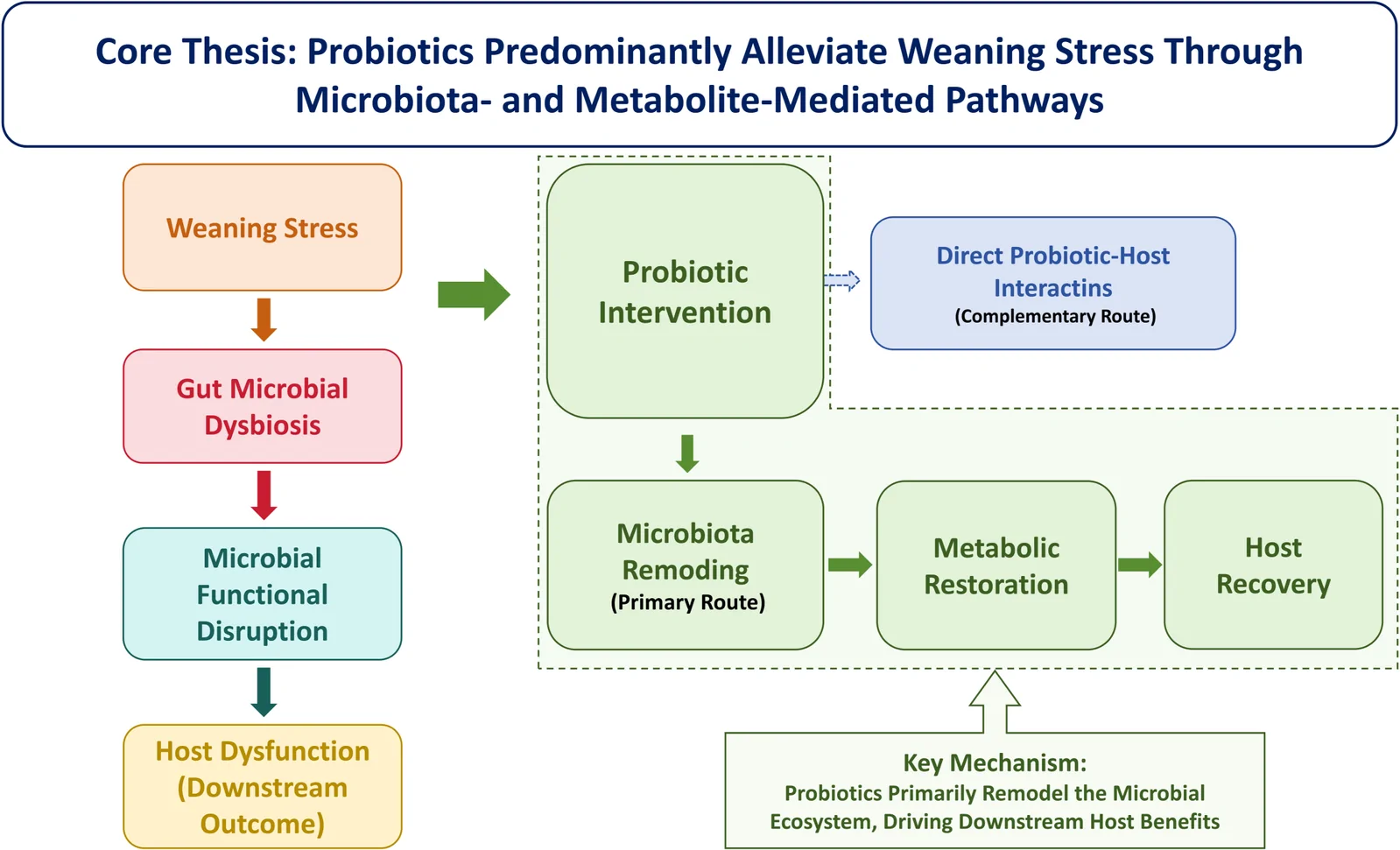
Conceptual framework of probiotic-mediated alleviation of weaning stress through microbiota- and metabolite-mediated pathways. Weaning stress disrupts the gut microbial ecosystem, resulting in microbial functional disruption and downstream host dysfunction. Probiotic intervention primarily promotes microbiota remodeling and metabolic restoration, ultimately contributing to host recovery, while direct probiotic-host interactions may provide complementary effects.

## 1 Introduction

Early weaning has become a routine management practice in modern swine production to enhance sow reproductive efficiency and improve overall production profitability (1). However, this management strategy markedly shortens the natural suckling period, which normally lasts 14-18 weeks, thereby exposing piglets to multiple physiological and environmental stressors at a stage when their digestive, immune, and neuroendocrine systems are still immature (2, 3). These stressors include the abrupt transition from highly digestible liquid milk to solid diets containing complex carbohydrates and anti-nutritional factors, behavioral stress associated with maternal separation, changes in housing conditions such as temperature, humidity, and stocking density, as well as regrouping with unfamiliar littermates (4, 5). Collectively, these challenges result in reduced feed intake, impaired growth performance, and a marked increase in the incidence of post-weaning diarrhea, which affects approximately 30-50% of weaned piglets. In severe cases, dehydration, electrolyte imbalance, and secondary infections can lead to mortality (6–8). In addition, weaning stress disrupts the composition and function of the intestinal microbiota, compromises intestinal barrier integrity, and suppresses both innate and adaptive immune responses, thereby predisposing piglets to a long-term health impairment that adversely affects subsequent growth and finishing performance (9, 10).

For decades, antibiotic growth promoters (AGPs) have been extensively incorporated into swine diets to control disease and maintain piglet health (11). Increasing evidence indicates that the extensive application of AGPs has substantially accelerated the emergence and spread of antimicrobial resistance (AMR), creating a major challenge for global public health (12, 13). Consequently, regulations restricting or prohibiting the use of AGPs in animal production have been progressively implemented worldwide. The European Union prohibited the non-therapeutic use of AGPs in animal feed in 2006, while China enacted a nationwide ban on antibiotic feed additives for livestock and poultry production in 2020 (14, 15). Under these circumstances, the development of safe, effective, and sustainable alternatives capable of maintaining intestinal health and mitigating weaning-associated disorders has become an urgent priority and a major scientific challenge for the swine industry.

Probiotics are live microorganisms that, when administered in adequate amounts, confer a health benefit on the host. As a promising alternative to AGPs, probiotics have attracted considerable attention because of their potential to improve animal health while reducing reliance on antibiotics and the associated risks of antimicrobial resistance and drug residues (16). Growing evidence indicates that the primary benefit of probiotics lies in their ability to modulate both the composition and function of the gut microbiota, thereby maintaining the dynamic equilibrium of the microbiota-host axis (17–19). Beyond supporting efficient nutrient digestion and absorption, a balanced gut microbiota is essential for maintaining intestinal barrier function and orchestrating appropriate host immune responses (18). During weaning, probiotic supplementation can suppress the excessive expansion of opportunistic pathogens, promote the establishment of beneficial commensal microorganisms, and exert multiple protective effects through the production of bioactive microbial metabolites (20, 21).

This review summarizes recent progress in understanding the application of probiotics for mitigating weaning stress in piglets. Particular attention is given to the molecular and cellular mechanisms by which probiotics reshape the gut microbiota, reinforce intestinal barrier integrity, regulate inflammatory pathways, and ultimately enhance growth performance. Recent applications of probiotics in weaned piglets are summarized, and their mechanisms of action, functional characteristics, and beneficial effects on the intestinal microbiota are discussed, with the goal of providing a scientific basis for future research and practical implementation in swine production systems.

## 2 Weaning stress-induced gut microbial dysbiosis in piglets

During the suckling period, the intestinal microbiota of piglets is relatively simple and stable, being predominantly composed of members of the phyla *Firmicutes* and *Bacteroidetes*, with *Lactobacillus* and *Bacteroides* serving as the dominant genera. This microbial configuration is well adapted to the lactose, oligosaccharides, and other nutrients provided by maternal milk, thereby supporting a relatively stable symbiotic relationship between the host and its gut microbiota (22–24). Following weaning, however, substantial changes in feed sources and nutrient composition alter the substrate landscape upon which the resident microbiota depends, disrupting the previously established microbial equilibrium. High-throughput sequencing studies have consistently identified weaning as a critical transition point in the developmental succession of the piglet gut microbiota (23, 25).

One of the earliest consequences of weaning is extensive remodeling of the intestinal microbial community. The abundance of *Lactobacillus* declines significantly, whereas members of the phylum *Proteobacteria* and the family *Enterobacteriaceae* expand markedly. Concurrent increases in the abundance of *Clostridium* spp. and *Prevotella* spp. have also been reported, indicating a shift from a commensal-dominated microbiota to one enriched in opportunistic pathogens (26, 27). Beyond compositional alterations, the normal developmental trajectory of the gut microbiota is also disrupted. The maturation process, which typically progresses from an early *Enterobacteriaceae*-dominated community toward one enriched in *Prevotellaceae* and *Ruminococcaceae*, may be delayed or diverted following weaning, resulting in prolonged microbial instability (28, 29).

Alterations in microbial composition and ecological succession are accompanied by substantial changes in microbial function (30). Evidence suggests that weaning induces extensive remodeling of key metabolic pathways, including carbohydrate metabolism, glycolysis, and the tricarboxylic acid (TCA) cycle. These alterations are accompanied by reduced production of short-chain fatty acids (SCFAs) and abnormal accumulation of intermediate metabolites such as succinate, indicating that the microbial metabolic network shifts from a homeostatic state toward a stress-adaptive configuration (27, 31). Furthermore, disruption of microbial metabolic activity may influence host immune and metabolic regulatory processes. Altered production of microbiota-derived metabolites has been linked to changes in host microRNA regulatory networks, including ssc-miR-425-5p and ssc-miR-423-3p, which may contribute to the development of post-weaning diarrhea (32, 33).

Taken together, weaning stress initiates a cascade of events by altering the intestinal nutritional environment, thereby driving changes in microbial community structure, developmental succession, and metabolic function. These interconnected alterations link microbial ecological disruption with downstream metabolic, immunological, and physiological dysfunction in the host (**Figure 1**). Consequently, gut microbial dysbiosis represents one of the earliest events in the pathogenesis of weaning-associated intestinal dysfunction and serves as a key driver of subsequent intestinal disorders.

**Figure 1:**
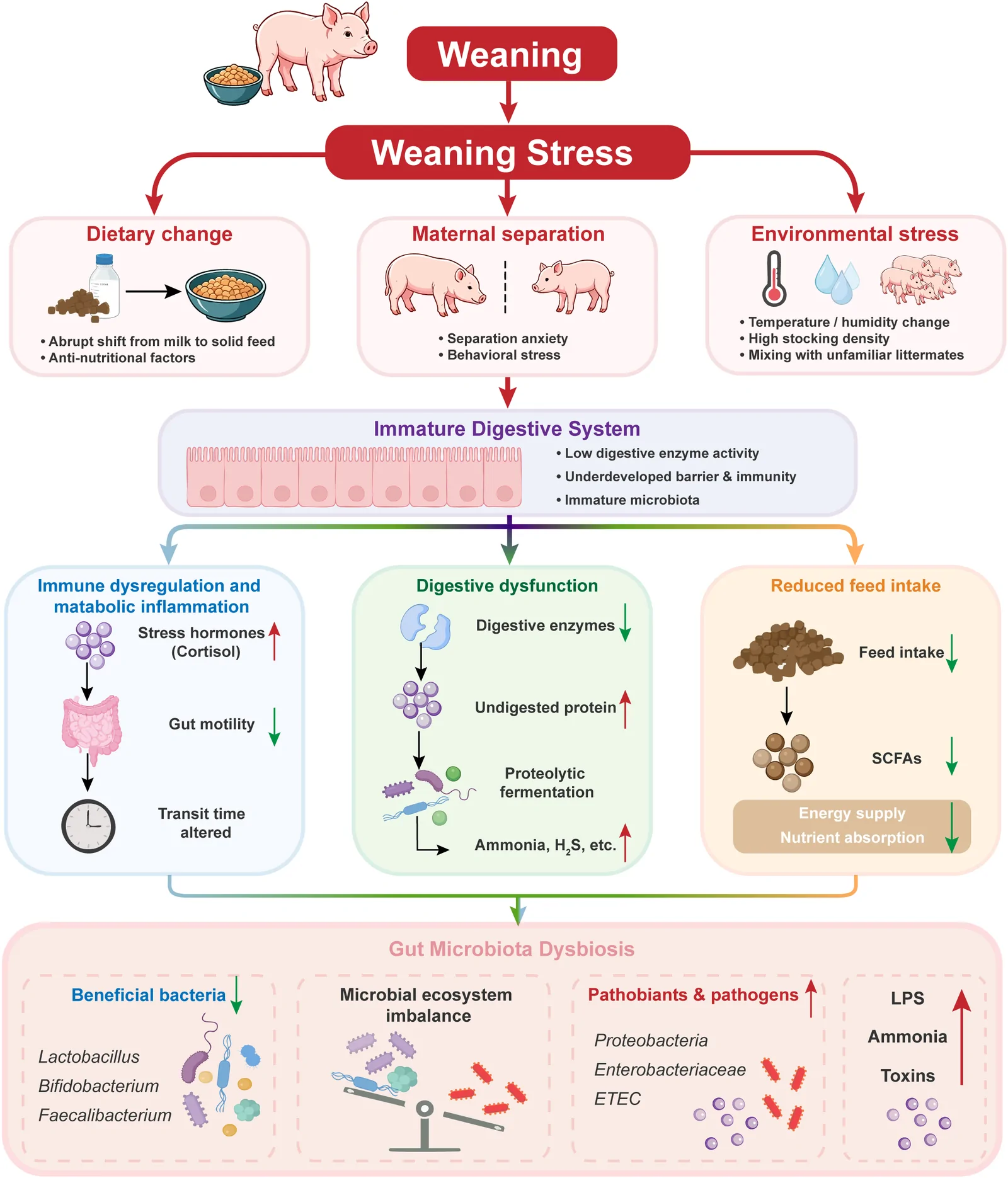
Weaning stress-induced gut microbial dysbiosis and its associated intestinal dysfunction in piglets. Weaning imposes multiple concurrent stressors, including abrupt dietary transition, maternal separation, and environmental changes, which act on the immature digestive and intestinal systems of piglets. These challenges contribute to impaired digestive function, altered intestinal motility and transit, reduced feed intake, and metabolic disturbances, including increased proteolytic fermentation and accumulation of potentially harmful metabolites such as ammonia and hydrogen sulfide. These changes reshape the intestinal microbial ecosystem, characterized by reduced beneficial bacteria, particularly *Lactobacillus*, *Bifidobacterium*, and *Faecalibacterium*, together with expansion of potentially pathogenic taxa, including *Proteobacteria*, *Enterobacteriaceae*, and enterotoxigenic *Escherichia coli* (ETEC). Microbial metabolic dysfunction is further associated with reduced short-chain fatty acid (SCFA) production and increased levels of lipopolysaccharide (LPS), ammonia, and other microbial-derived toxic metabolites. Collectively, these compositional, ecological, and metabolic alterations disrupt intestinal homeostasis and provide a mechanistic link between weaning stress and subsequent intestinal dysfunction in piglets. ↑, increased; ↓, decreased.

## 3 Consequences of gut microbiota dysbiosis: disruption of host homeostasis

The intestinal microbiota is a key regulator of gastrointestinal homeostasis and participates extensively in essential physiological processes, including nutrient metabolism, barrier maintenance, immune regulation, and redox balance. Beyond altering microbial community composition and metabolic function, weaning stress-induced dysbiosis acts as an upstream driver of multiple pathological responses in the host. Increasing evidence indicates that dysbiosis represents a critical mechanistic link between weaning stress and intestinal dysfunction. The pathological consequences of dysbiosis include disruption of intestinal barrier integrity, immune dysregulation, oxidative stress, and metabolic inflammation. Together, these interconnected processes contribute to the pathophysiological basis of post-weaning intestinal injury (**Figure 2**) (8, 26, 34).

**Figure 2:**
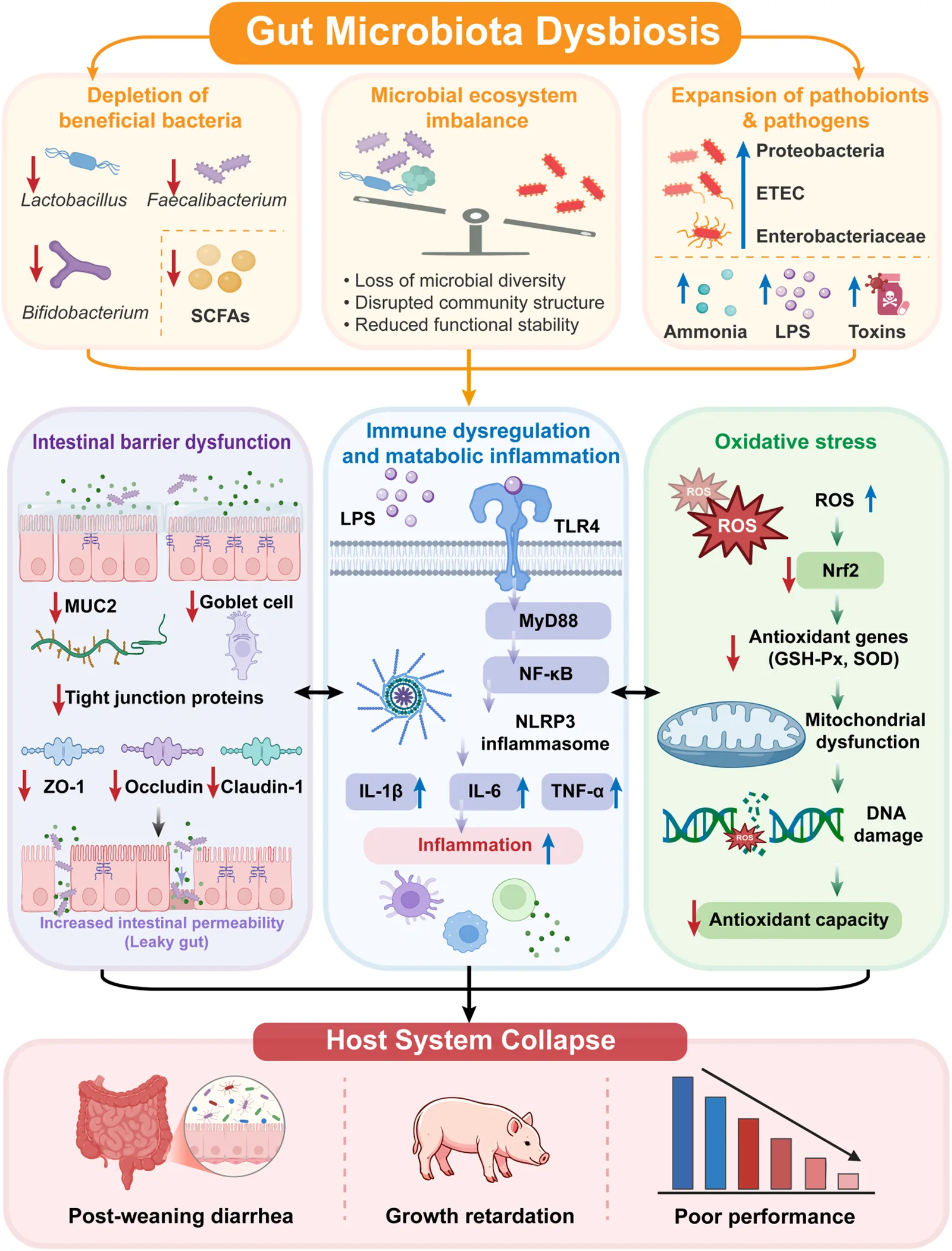
Pathological consequences of gut microbial dysbiosis in weaning piglets. Weaning-associated gut microbial dysbiosis is characterized by depletion of beneficial bacteria and SCFA production, disruption of microbial community structure and functional stability, and expansion of pathobionts and pathogens. These microbial alterations converge on three interconnected pathological pathways. (i) Intestinal barrier dysfunction: reduced SCFA availability compromises mucus production and goblet cell function, decreases the expression of tight junction proteins, including ZO-1, occludin, and claudin-1, and consequently increases intestinal permeability. (ii) Immune dysregulation and metabolic inflammation: increased microbe-associated molecular patterns (MAMPs), particularly lipopolysaccharide (LPS), activate TLR4-MyD88-NF-κB signaling and the NLRP3 inflammasome, promoting the production of pro-inflammatory cytokines, including IL-1β, IL-6, and TNF-α, and potentially facilitating systemic endotoxemia. (iii) Oxidative stress: dysbiosis-associated metabolic disturbances increase reactive oxygen species (ROS) generation, impair antioxidant defenses, including SOD and GSH-Px, and disrupt Nrf2 signaling, thereby promoting mitochondrial dysfunction and oxidative damage. Crosstalk among these pathways further amplifies intestinal inflammation and tissue injury, ultimately contributing to post-weaning diarrhea, growth retardation, and impaired growth performance. Thus, gut microbial dysbiosis represents a central mechanistic link between weaning stress and the collapse of intestinal and systemic homeostasis. ↑, increased; ↓, decreased. GSH-Px, glutathione peroxidase; LPS, lipopolysaccharide; MAMPs, microbe-associated molecular patterns; MyD88, myeloid differentiation primary response 88; Nrf2, nuclear factor erythroid 2-related factor 2; ROS, reactive oxygen species; SCFAs, short-chain fatty acids; TLR4, Toll-like receptor 4; ZO-1, zonula occludens-1.

### 3.1 Intestinal barrier dysfunction

Acting as the first physical barrier against enteric pathogens, the intestinal epithelium depends on the coordinated maintenance of tight junction complexes, the mucus layer, and commensal microbiota. Under physiological conditions, commensal bacteria ferment dietary carbohydrates to produce SCFAs, which play a central role in maintaining intestinal barrier homeostasis. Butyrate, in particular, serves as the preferred energy source for colonocytes, whereas acetate and propionate are more extensively metabolized systemically. Collectively, SCFAs promote mucin secretion and tight junction protein expression, thereby supporting epithelial integrity and preserving intestinal barrier function (35, 36).

Following weaning, the abundance of beneficial microorganisms such as *Lactobacillus* and *Bifidobacterium* declines substantially, resulting in reduced SCFA production. Consequently, the mucus layer becomes thinner, and energy availability for intestinal epithelial cells is reduced. Simultaneously, dysbiosis is accompanied by the expansion of opportunistic pathogens and the accumulation of pro-inflammatory microbial metabolites, which further destabilize tight junction structures, increase intestinal permeability, and facilitate bacterial translocation (26, 35, 37, 38). Studies have demonstrated that weaned piglets commonly exhibit villus atrophy, crypt hyperplasia, and reduced expression of tight junction proteins, including zonula occludens-1 (ZO-1), occludin, and claudin-1, indicating significant impairment of intestinal barrier function (39, 40). Therefore, intestinal barrier disruption is widely regarded an important initiating event linking microbial dysbiosis to pathological alterations in the host.

### 3.2 Immune dysregulation and metabolic inflammation

The establishment and long-term maintenance of mucosal immunity are highly dependent on continuous interactions between the host and its intestinal microbiota. Under homeostatic conditions, commensal microorganisms and their metabolites promote the differentiation of regulatory T cells (Tregs) and maintain the dynamic balance between Treg and T helper 17 (Th17) cells, thereby establishing an appropriate state of immune tolerance (41, 42). Weaning stress alters both microbial composition and microbial ecological interactions, leading to impaired immunomodulatory capacity within the intestine. Under these conditions, microbe-associated molecular patterns (MAMPs) generated during dysbiosis continuously stimulate pattern recognition receptors. Among these receptors, Toll-like receptor 4 (TLR4) is considered a key molecular bridge linking microbial alterations to host immune responses (43). Activation of TLR4 subsequently initiates both the MyD88-dependent pathway, which mediates the rapid activation of nuclear factor kappa B (NF-κB) and mitogen-activated protein kinase (MAPK) signaling, and the MyD88-independent/TRIF-dependent pathway. The TRIF-dependent pathway activates IRF3 to induce type I interferon production and also contributes to a delayed NF-κB and MAPK activation. These pathways collectively orchestrate the downstream inflammatory response, leading to the transcriptional upregulation of pro-inflammatory cytokines such as interleukin-1β (IL-1β), IL-6, and tumor necrosis factor-α (TNF-α) (44, 45). This process perpetuates local inflammatory responses and consequently disrupts immune homeostasis (8, 46, 47).

Concurrently, alterations in microbial composition are accompanied by profound metabolic reprogramming, particularly a marked reduction in SCFA production. SCFA depletion reduces their protective effects on epithelial energy metabolism, barrier maintenance, and immune regulation, thereby further amplifying inflammatory responses (36, 48). In contrast, the expansion of *Proteobacteria* and members of the family *Enterobacteriaceae* increases intestinal lipopolysaccharide (LPS) levels. Following translocation across the compromised epithelial barrier, LPS enters the mucosa and systemic circulation, where it sustains inflammatory signaling through TLR4, thereby contributing to metabolic endotoxemia (35, 47, 49). Moreover, weaning-associated dysbiosis promotes the abnormal accumulation of microbial metabolic intermediates, such as succinate. These metabolites further participate in inflammatory amplification and contribute to the development of diarrheal phenotypes (31). Collectively, SCFA depletion and LPS accumulation constitute two major driving forces underlying post-weaning immune-metabolic dysregulation.

### 3.3 Oxidative stress: the ROS-Nrf2 axis

Gut microbiota dysbiosis also exerts profound effects on host redox homeostasis. Studies have demonstrated that alterations in microbial composition and metabolic profiles following weaning promote excessive reactive oxygen species (ROS) production and are frequently accompanied by mitochondrial dysfunction (48, 50). Meanwhile, the antioxidant defense system of weaned piglets remains immature, as evidenced by reduced activities of key antioxidant enzymes, including superoxide dismutase (SOD) and glutathione peroxidase (GSH-Px), thereby limiting the capacity for ROS scavenging (10, 51). Excessive ROS accumulation induces lipid peroxidation, protein oxidation, and DNA damage while simultaneously overwhelming or dysregulating the nuclear factor erythroid 2-related factor 2 (Nrf2)-mediated antioxidant defense system, further aggravating oxidative injury (50, 52). Therefore, excessive ROS generation coupled with compromised Nrf2-dependent protective mechanisms represents a hallmark feature of oxidative stress under weaning conditions.

In summary, weaning-induced gut microbiota dysbiosis not only results in profound alterations in microbial community structure and function but also acts as an upstream pathological driver that initiates intestinal barrier disruption, immune dysregulation, redox imbalance, and metabolic inflammation. Through these interconnected pathological processes, intestinal dysfunction following weaning is synergistically exacerbated. Overall, TLR4 functions as a critical molecular bridge linking microbial alterations to host inflammatory responses, whereas the NF-κB signaling axis, ROS-Nrf2 pathway, and SCFA signaling axis constitute key regulatory modules governing immune inflammation, oxidative stress homeostasis, and energy metabolism, respectively. Collectively, these interconnected pathways establish a continuous pathogenic cascade linking gut microbial dysbiosis to immune and metabolic dysfunction, ultimately leading to impaired intestinal homeostasis and the progression of weaning-associated intestinal injury.

## 4 Restoration of intestinal homeostasis through probiotic-mediated remodeling of the gut microecosystem

From a microbial ecological perspective, probiotics may act as “ecosystem engineers” by altering niche availability, resource competition, and microbial interactions, thereby reshaping community assembly and the competitive structure of the gut microbiota. They may also facilitate metabolic cross-feeding among microbial taxa, linking microbial community remodeling to the recovery of functional metabolites and downstream host responses.

In response to weaning stress, probiotic supplementation may contribute to the maintenance of intestinal homeostasis through interactions with the resident microbial ecosystem and, to a lesser extent, through direct probiotic-host interactions. MAMPs, including lipoteichoic acid, peptidoglycan, surface-layer proteins, and exopolysaccharides, can be recognized by pattern recognition receptors (PRRs) on intestinal epithelial and immune cells, thereby modulating TLR2, NOD-like receptor, and downstream MyD88-NF-κB and MAPK signaling pathways. These interactions may influence the expression of inflammatory cytokines, antimicrobial peptides, and tight junction proteins, thereby contributing to intestinal barrier integrity and mucosal immune homeostasis (53–55). In weaned piglets, specific probiotic strains may also directly promote epithelial barrier repair. For example, *Lactobacillus frumenti* supplementation increased the expression of ZO-1, occludin, and claudin-1 and improved intestinal mucosal integrity in early-weaned piglets (56). However, these direct probiotic-host interactions are highly strain-specific and depend on the surface characteristics of the probiotic strain, host receptor expression, and the local intestinal environment. Moreover, direct probiotic effects on host cells are closely intertwined with changes in the gut microbial ecosystem. Accumulating evidence indicates that probiotics can reshape microbial community composition and function, thereby altering the production of microbiota-derived metabolites such as short-chain fatty acids, bile acids, and tryptophan-derived metabolites, which can subsequently function as signaling mediators to regulate intestinal barrier integrity and immune homeostasis (57, 58). Thus, while direct probiotic-host signaling represents an important complementary mechanism, microbiota remodeling and restoration of microbial metabolic functions may represent an important route linking probiotic intervention to host homeostasis (**Figure 3**).

**Figure 3:**
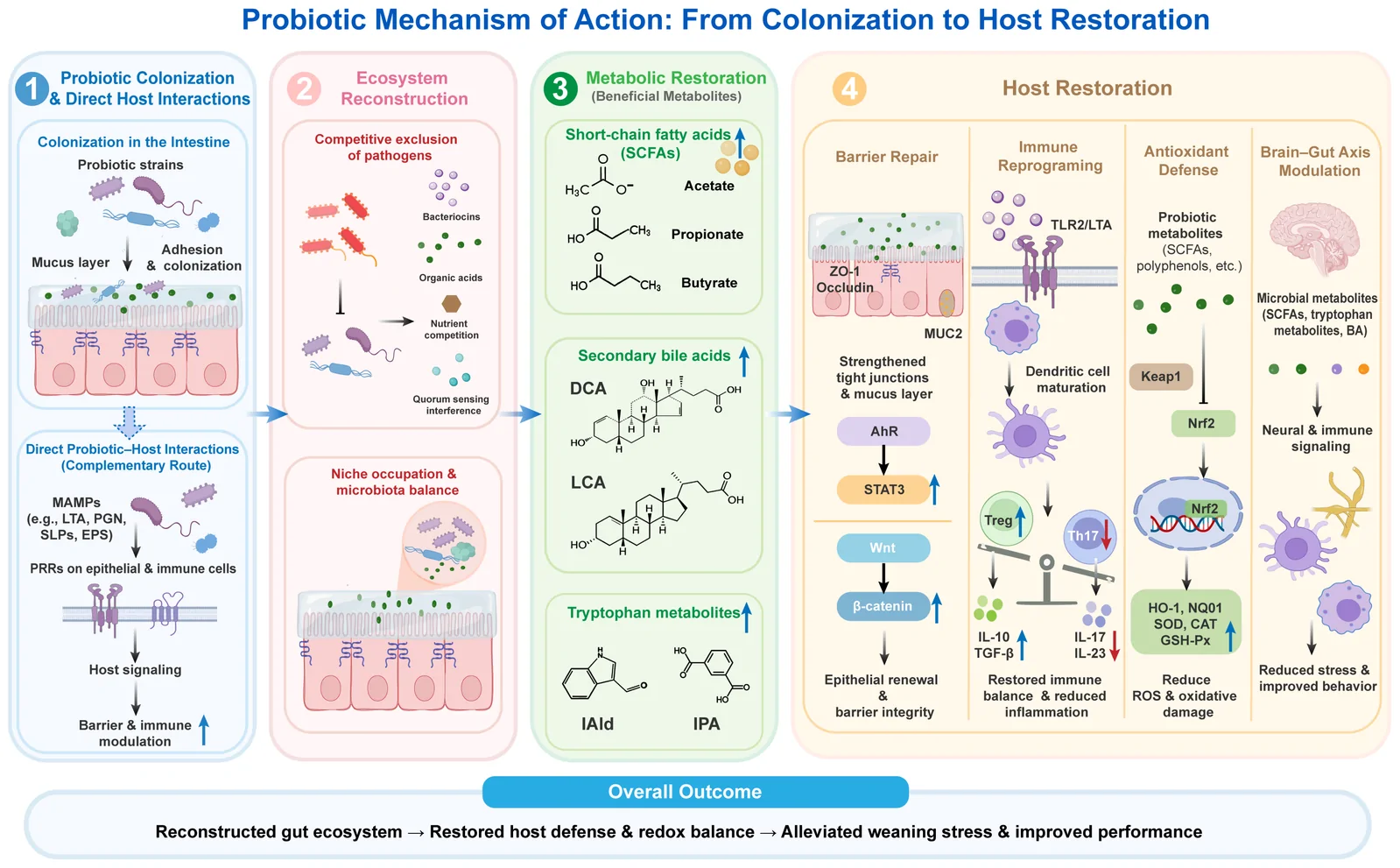
Probiotic mechanism of action from microbial colonization to host restoration. Probiotics primarily act on the intestinal microbial ecosystem through a sequential process involving probiotic colonization, ecosystem reconstruction, metabolic restoration, and subsequent host functional recovery (1). Probiotic colonization and direct host interactions: probiotic strains adhere to the intestinal mucus layer and establish colonization, while complementary direct interactions with epithelial and immune cells may occur through microbial-associated molecular patterns (MAMPs) and pattern-recognition receptors (PRRs), modulating host signaling and barrier and immune responses. (2) Ecosystem reconstruction: probiotic colonization promotes competitive exclusion of pathogens through nutrient competition, production of organic acids and bacteriocins, quorum-sensing interference, and niche occupation, thereby contributing to restoration of microbial community balance. (3) Metabolic restoration: remodeling of the gut microbial ecosystem enhances the production of beneficial metabolites, including short-chain fatty acids (SCFAs; acetate, propionate, and butyrate), secondary bile acids (e.g., DCA and LCA), and tryptophan-derived metabolites (e.g., indole-3-aldehyde [IAld] and indole-3-propionic acid [IPA]). (4) Host restoration: microbiota- and metabolite-mediated signals contribute to host recovery through four interconnected pathways, including (i) barrier repair, involving reinforcement of the mucus layer and tight junctions and promotion of epithelial renewal; (ii) immune reprogramming, involving modulation of microbial pattern-recognition signaling and restoration of immune balance; (iii) antioxidant defense, involving activation of the Keap1-Nrf2 pathway enhancement of antioxidant defenses; and (iv) brain-gut axis modulation, involving microbial metabolite-mediated neural and immune signaling and associated improvements in stress responses and behavior. Collectively, probiotic-driven ecosystem reconstruction restores intestinal barrier integrity, immune homeostasis, redox balance, and brain-gut function, thereby alleviating weaning-associated intestinal dysfunction and improving growth performance. The schematic emphasizes that probiotics exert their beneficial effects predominantly through remodeling the gut microbial ecosystem and its metabolic functions, whereas direct probiotic-host interactions represent a complementary route. AhR, aryl hydrocarbon receptor; CAT, catalase; DCA, deoxycholic acid; GSH-Px, glutathione peroxidase; HO-1, heme oxygenase-1; IAld, indole-3-aldehyde; IPA, indole-3-propionic acid; Keap1, Kelch-like ECH-associated protein 1; LCA, lithocholic acid; LTA, lipoteichoic acid; NQO1, NAD(P)H quinone dehydrogenase 1; Nrf2, nuclear factor erythroid 2-related factor 2; SCFAs, short-chain fatty acids; STAT3, signal transducer and activator of transcription 3; Treg, regulatory T cell; Th17, T helper 17 cell; Wnt, Wingless-related integration site; ZO-1, zonula occludens-1.

### 4.1 Reconstruction of the gut microecosystem

Weaning stress initially disrupts the composition of the gut microbiota, impairs microbial community maturation, and promotes the expansion of opportunistic pathogens, ultimately disturbing intestinal microecological homeostasis. Increasing evidence suggests that the primary target of probiotic action in alleviating weaning stress may be mediated through the intestinal microbial ecosystem rather than direct actions on host tissues (59–61). According to community assembly theory, microbial communities are shaped by deterministic ecological selection together with stochastic ecological processes. Probiotic intervention may influence these processes by altering niche availability, resource competition, and microbial interactions, thereby favoring health-associated microbial populations while constraining the expansion of opportunistic pathogens (62–64). On the one hand, probiotics such as *Lactobacillus*, *Bifidobacterium*, and *Bacillus* species can preferentially occupy epithelial adhesion sites through competitive exclusion and niche occupation and compete for critical nutrients, including oligosaccharides, amino acids, and iron, thereby reducing the colonization capacity of enterotoxigenic *Escherichia coli* (ETEC), enteropathogenic *E. coli* (EPEC), and other members of the family *Enterobacteriaceae* (65–70). Following gastrointestinal administration, probiotic strains must survive the gastrointestinal tract to exert their effects (71). However, survival, transient persistence, and stable engraftment represent distinct processes, and persistent colonization is not necessarily required for probiotic efficacy (72). Transiently persisting probiotics may still exert functional effects through competitive exclusion, antimicrobial metabolite production, and interactions with resident microbiota (73). Thus, functional activity rather than long-term engraftment may be a more relevant determinant of probiotic efficacy. On the other hand, probiotics facilitate microbial succession toward a more mature and stable community structure by increasing the abundance of beneficial taxa such as *Lactobacillus* and members of the family *Ruminococcaceae* while restraining the abnormal expansion of *Proteobacteria* (59–61). This ecological remodeling serves as the foundation for subsequent probiotic-mediated effects on metabolic regulation, immune modulation, and intestinal barrier function, thereby potentially contributing to the alleviation of weaning-associated intestinal dysfunction.

### 4.2 Restoration of microbial metabolic function

The biological functions of the gut microbiota extend beyond community composition and are largely mediated by microbiota-derived metabolites that regulate host physiology. Weaning stress induces extensive remodeling of microbial metabolic networks, characterized by reduced production of key functional metabolites, including SCFAs, secondary bile acids, and tryptophan-derived metabolites (74–78). Consequently, the regulatory capacity of microbiota over host homeostasis is substantially impaired. By promoting the restoration the intestinal microecological structure, probiotics may promote metabolic cross-feeding among microbial taxa and facilitate the recovery of these metabolic functions, providing a potential mechanistic link between microbial reconstruction and intestinal functional recovery (75–77, 79).

#### 4.2.1 Short-chain fatty acids

SCFAs, primarily acetate, propionate, and butyrate, are the principal end products of microbial fermentation of dietary fibers and complex carbohydrates. Studies have demonstrated that probiotics supplementation can increase the abundance of SCFA-associated taxa, including *Lactobacillus*, members of the family *Ruminococcaceae*, thereby promoting SCFA production (76, 80). As key metabolic mediators within the intestinal ecosystem, SCFAs reduce luminal pH, creating an acidic microenvironment that suppresses the growth of many Gram-negative pathogens and disrupts their energy metabolism and intracellular pH homeostasis (81, 82). In addition, SCFAs can enhance hypoxia-inducible factor-1 (HIF-1) signaling, thereby promoting antimicrobial peptide expression and supporting innate immune defense against pathogens (83).

Notably, butyrate functions not only as a major energy source for colonocytes but also as a potent signaling molecule. By inhibiting histone deacetylase (HDAC) activity, butyrate regulates host gene expression, while activation of the G protein-coupled receptors GPR41 and GPR43 mediates microbiota-host communication and contributes to the maintenance of intestinal homeostasis (76, 84, 85). Consequently, SCFAs are widely recognized as one of the most important metabolic mediators responsible for the host-protective effects of probiotics.

#### 4.2.2 Secondary bile acids

Secondary bile acids are generated through microbial biotransformation of primary bile acids secreted by the liver, with deoxycholic acid (DCA) and lithocholic acid (LCA) representing the most prominent metabolites (75). Weaning stress reduces the abundance of bile acid-transforming bacteria, thereby impairing secondary bile acid production. Probiotic supplementation promotes the recovery of these functional microbial populations, restores bile acid metabolism, and increases the levels of bioactive metabolites such as DCA and LCA (75, 86).

Beyond their established role in lipid metabolism, secondary bile acids also exert important ecological and host-regulatory functions within the gut. At sufficiently high local concentrations, secondary bile acids can directly disrupt bacterial membranes and inhibit pathogen growth, potentially contributing to intestinal colonization resistance (86–88). However, under physiological conditions, their protective effects are not limited to direct antimicrobial activity but are largely mediated through their roles as host signaling molecules. In particular, secondary bile acids activate the farnesoid X receptor (FXR) and G protein-coupled bile acid receptor 1 (TGR5), thereby promoting antimicrobial peptide production, modulating intestinal immune responses, and maintaining mucosal homeostasis and immune tolerance (89–91). Therefore, restoration of bile acid metabolism may represent considered an important mechanism through which probiotics facilitate microbiota-host communication and support intestinal homeostasis.

#### 4.2.3 Tryptophan-derived metabolites

Tryptophan metabolism represents a critical signaling interface between the gut microbiota and the host. Various probiotic and commensal microorganisms can metabolize dietary tryptophan into bioactive compounds, including indole-3-aldehyde (IAld), indole-3-propionic acid (IPA), and indole-3-carboxaldehyde (77, 92). Recent studies have demonstrated that these indole derivatives not only contribute to microbial community stability but also function as important molecular mediators linking the microbiota with host mucosal signaling networks (93, 94). Mechanistically, indole-derived metabolites act as endogenous ligands of the aryl hydrocarbon receptor (AhR), activating AhR-dependent signaling pathways and subsequently inducing the expression of mucosal protective factors (95–97). In addition, these metabolites modulate host adaptive responses to intestinal microorganisms and promote restoration of intestinal homeostasis within the gut (98–100). Therefore, the tryptophan metabolic pathway is regarded as an important component of probiotic-mediated microbiota-host communication.

Collectively, probiotics not only restore microbial composition but also re-establish key functional metabolic pathways, including SCFA, secondary bile acid, and tryptophan metabolism. These microbiota-derived metabolites constitute a central metabolic network linking microbial ecological remodeling with intestinal and systemic host responses.

### 4.3 Barrier protection

The intestinal barrier represents the primary interface between the microbiota and the host. Weaning stress-induced dysbiosis and metabolic dysfunction contribute to thinning of the mucus layer, reduced expression of tight junction proteins, and increased intestinal permeability (21, 101–104).

By restoring microbial metabolic activity, probiotics significantly improve intestinal barrier integrity. SCFAs, particularly butyrate, promote the expression of tight junction proteins such as ZO-1, occludin, and claudin-1, thereby strengthening epithelial intercellular junctions and reducing paracellular permeability (85, 102, 104). In parallel, probiotics stimulate goblet cell differentiation and MUC2 secretion, resulting in enhanced mucus layer thickness and continuity (105–109). Furthermore, certain probiotic strains activate signaling pathways such as AhR/STAT3 and Wnt/β-catenin, thereby promoting intestinal stem cell proliferation and epithelial renewal and accelerating the repair of damaged mucosa (105, 106, 108). Through these complementary mechanisms, including enhancement of epithelial regeneration, reinforcement of tight junction integrity, and restoration of the mucus barrier, probiotics effectively recover intestinal barrier function in weaned piglets.

### 4.4 Immune reprogramming

Weaning stress-induced dysbiosis continuously stimulates pattern recognition receptors, leading to disruption of immune homeostasis and persistent activation of pro-inflammatory responses. In contrast, probiotics may modulate host immune responses through remodeling of the gut microbiota and intestinal metabolic environment. In addition to microbiota-mediated effects, certain probiotic-derived microbial components can directly interact with host immune cells. For example, lipoteichoic acid (LTA) from specific probiotic strains can activate TLR2-mediated signaling, promoting the differentiation of dendritic cells toward a tolerogenic phenotype and enhancing IL-10 production (110, 111). This promotes the expansion of Tregs, the secretion of IL-10, and the production of transforming growth factor-β (TGF-β), thereby suppressing excessive Th1- and Th17-mediated immune responses (41, 112).

Meanwhile, probiotics inhibit the activation of inflammatory signaling pathways, including TLR4/MyD88/NF-κB and MAPK, resulting in reduced expression of pro-inflammatory cytokines such as TNF-α, IL-1β, and IL-6 (60, 61, 113, 114). In weaned piglet models, supplementation with *Clostridium butyricum*, *Lactobacillus johnsonii*, and multi-strain probiotic formulations has been reported to improve intestinal immune homeostasis and attenuate inflammatory injury (59–61, 115).

### 4.5 Attenuation of oxidative stress

Oxidative stress is a major amplifying factor in post-weaning intestinal injury. Dysbiosis promotes excessive generation of ROS, while the immature antioxidant defense system of newly weaned piglets is unable to efficiently neutralize excessive ROS, ultimately leading to mitochondrial dysfunction and epithelial damage (116, 117). Probiotics alleviate oxidative stress by improving microbial metabolic function and enhancing host antioxidant defenses. Numerous studies have demonstrated that lactic acid bacteria, yeasts, and *Bacillus* species increase the activities of superoxide dismutase (SOD), catalase (CAT), and GSH-Px, while reducing ROS and malondialdehyde (MDA) levels (118–121).

Mechanistically, Nrf2 is considered a central regulator of the antioxidant effects of probiotics. Probiotics activate the Nrf2-Keap1 signaling axis, promoting the expression of antioxidant genes such as heme oxygenase-1 (HO-1) and NAD(P)H quinone dehydrogenase 1 (NQO1), thereby enhancing cellular antioxidant capacity and preserving mitochondrial homeostasis (122, 123). The attenuation of oxidative stress subsequently facilitates barrier repair and resolution of inflammation, thereby establishing a positive feedback loop that further promotes restoration of intestinal homeostasis.

### 4.6 Modulation of the gut-brain axis

Beyond local intestinal effects, probiotics may influence systemic responses to weaning stress through the gut-brain axis. Microbiota-derived metabolites, including SCFAs and tryptophan-derived indoles, can interact with enteroendocrine cells and vagal afferent pathways, thereby influencing neuroendocrine signaling and stress-related responses (124–126). These gut-brain interactions may also affect appetite and feeding behavior through gut-derived hormonal and neural signals (127). In weaned piglets, probiotic supplementation has been associated with changes in hypothalamic neurotransmitters alongside improvements in intestinal and growth-related outcomes, providing preliminary evidence for gut-brain communication during the post-weaning period (128). Although direct evidence in weaned piglets remains limited, emerging findings suggest that probiotic-mediated remodeling of the gut microbiota and its metabolic outputs may contribute to the regulation of systemic stress responses beyond the intestinal compartment. Further studies are needed to clarify the contribution of specific microbial metabolites and neural or neuroendocrine pathways to these effects.

## 5 Common probiotic strains for alleviating weaning stress through gut microbiota modulation

With the gradual withdrawal of AGPs from swine production systems, probiotics have emerged as one of the most promising alternatives for mitigating weaning stress in piglets due to their high safety profile, low risk of inducing antimicrobial resistance, and sustained ability to regulate the gut microbial ecosystem (**Table 1**) (129, 130). Currently, the most widely used probiotics include lactic acid bacteria (LAB), *Bacillus* spp., and yeasts. Although these microorganisms differ in their biological characteristics and strain-specific mechanisms of action, their reported effects may converge on modulation of the gut microbial ecosystem, restoration of microbial metabolic function, and improving of host physiological outcomes (8, 26, 130, 131).

**Table 1:** Strain-specific evidence for probiotic interventions in alleviating weaning stress in piglets through modulation of the gut microbiota.

Probiotic category	Specific strain(s)/formulation	Microbiota remodeling	Mechanistic evidence	Major beneficial effects	References
Lactic acid bacteria	*Lactobacillus frumenti*	Increases lactobacilli abundance and reduced potential pathogenic bacteria	Activated AMPK signaling and increased ZO-1 and occludin expression	Improves intestinal barrier integrity	(56)
Lactic acid bacteria	*Lactobacillus delbrueckii*	Increased the abundance of *Blautia* and *Ruminococcaceae*	Enhanced SCFA production	Increases villus height and improves growth performance	(133)
Multi-strain probiotic formulation	*Lactobacillus* sp. *+ Bifidobacterium thermacidophilum*	Optimizes cecal microbial composition	Modulated cecal microbial composition and promoted microbial homeostasis	Improves average daily gain (ADG) and reduces inflammation	(134)
Multi-strain probiotic combination	*Enterococcus faecalis + Clostridium butyricum*	Increases the abundance of butyrate-producing bacteria	Reduced TLR4/MyD88/NF-κB signaling and attenuated LPS-induced inflammation	Attenuates LPS-induced inflammation	(136)
Lactic acid bacteria	*Pediococcus pentosaceus*	Reduces the abundance of potential pathogens	Suppresses NF-κB signaling, upregulates tight junction protein expression, and enhances fecal microbial diversity	Alleviates inflammation and improves feed efficiency	(182)
*Bacillus* spp.	*Bacillus subtilis*	Modulates tryptophan-metabolizing microbial populations	Modulated tryptophan metabolism and the AhR signaling axis	Enhances immune function	(141)
*Bacillus* spp.	*Bacillus licheniformis*	Increases *Lactobacillus* abundance	Increased intestinal IgA secretion	Reduces the incidence of diarrhea	(140)
*Bacillus* spp.	*Bacillus amyloliquefaciens*	Maintains microbial homeostasis	Modulated microbial homeostasis and increased SOD and GSH-Px activities	Mitigates oxidative stress	(142)
Multi-strain *Bacillus* preparation	*B. subtilis + B. licheniformis + B. amyloliquefaciens*	Increases microbial diversity	Microbiota remodeling and immune regulation	Improves intestinal morphology and immune function	(60)
Yeasts	*Saccharomyces cerevisiae*	Promotes the proliferation of *Lactobacillus* and *Bifidobacterium*	Potential pathogen exclusion associated with MOS and β-glucans	Improves the intestinal microecological environment	(148, 149)
Yeasts	*Iron-enriched Saccharomyces cerevisiae*	Increases the abundance of lactobacilli	Enhances antioxidant enzyme activities	Alleviates oxidative stress	(155)
Yeast blend	*S. cerevisiae and related strains*	Increasing the abundance of beneficial bacteria	Improves the intestinal microenvironment	Increases villus height	(154)
Multi-strain probiotic formulation	*LAB + Bacillus* spp. *+ Yeasts*	Increases SCFA production and microbial diversity	Microbiota remodeling combined with immune modulation	Improves growth performance and reduces diarrhea incidence	(157)

SCFAs, short-chain fatty acids; AMPK, AMP-activated protein kinase; AhR, aryl hydrocarbon receptor; IgA, immunoglobulin A; SOD, superoxide dismutase; GSH-Px, glutathione peroxidase; MOS, mannan oligosaccharides; LAB, lactic acid bacteria; LPS, lipopolysaccharide.

### 5.1 Lactic acid bacteria

LAB are among the most extensively studied probiotic groups in weaned pig production, with commonly investigated genera including *Lactobacillus*, *Enterococcus*, and *Pediococcus*. *Bifidobacterium* species are often used alongside LAB but are taxonomically distinct (130). Studies of specific LAB strains have reported enrichment of health-associated bacterial taxa, including *Lactobacillus*, *Bifidobacterium*, and members of the family Ruminococcaceae, together with reductions in potential opportunistic pathogens, thereby enhancing microbial stability after weaning (56, 132, 133).

For example, *Lactobacillus delbrueckii* significantly increases the abundance of *Blautia* and Ruminococcaceae, whereas supplementation with *Lactobacillus* spp. combined with *Bifidobacterium thermacidophilum* optimizes cecal microbial composition and enhances microbial diversity (133, 134). In specific probiotic interventions, LAB-associated microbiota remodeling has been accompanied by restoration of SCFA production. Increased abundance of butyrate- and propionate-producing bacteria may contribute to enhanced intestinal fermentation and increased butyrate availability, resulting in a more favorable intestinal metabolic profile (135, 136). Restoration of microbial composition and metabolic functions has been associated with improved villus development, growth performance, reduced diarrhea incidence, and attenuated intestinal inflammation. Specifically, *Lactobacillus frumenti* has been shown to improve intestinal integrity and growth performance (56, 137), whereas *Lactobacillus delbrueckii* promotes villus development and increases average daily gain (133). Multi-strain LAB formulations have also been reported to simultaneously enhance immune function and productive performance (134).

### 5.2 *Bacillus* species

Due to their ability to form highly resistant endospores, *Bacillus* species exhibit excellent tolerance to feed pelleting, elevated temperatures, and storage conditions, making them among the most widely commercialized probiotics in animal production (129, 131). Supplementation with specific *Bacillus* strains or multi-strain formulations increases microbial diversity in the intestine of weaned piglets, promotes the colonization of beneficial bacteria such as *Lactobacillus*, and suppresses the proliferation of potential pathogens (138, 139). For instance, *Bacillus licheniformis* significantly increases the abundance of lactobacilli, while a three-strain *Bacillus* consortium improves overall microbial community structure and strengthens microbial network stability (60, 140). Studies have shown that *Bacillus subtilis* reshapes the intestinal tryptophan metabolic network and enhances the production of related metabolites (141), whereas *Bacillus amyloliquefaciens* improves the intestinal fermentation environment and enhances antioxidant capacity (142). Moreover, some multi-strain *Bacillus* formulations have been reported to enhance nutrient degradation and utilization, thereby improving intestinal metabolic efficiency (143, 144). Across studies of *Bacillus* strains and formulations, reported benefits include reduced post-weaning diarrhea, improved growth performance, and enhanced intestinal or immune function (145, 146). In addition, multispecies *Bacillus* spore-based formulations simultaneously improve growth performance and immune function in weaned piglets (60).

### 5.3 Yeasts and yeast-derived products

Yeast-based probiotics mainly include *Saccharomyces cerevisiae* strains, whereas yeast-derived or non-viable components, such as mannan oligosaccharides (MOS) and β-glucans, are considered separately because they do not constitute live probiotic microorganisms (147). Although some administered yeast strains may exhibit limited long-term colonization capacity within the gastrointestinal tract, specific yeast preparations have been reported to modulate the intestinal microbial ecosystem, thereby promoting the proliferation of beneficial bacteria such as *Lactobacillus* and *Bifidobacterium* while reducing pathogen colonization (26, 148, 149).

Yeast-based formulations have been shown to significantly increase the proportion of beneficial microorganisms and optimize microbial community structure in weaned piglets (150). Their reported benefits may involve changes in nutrient utilization and microbial metabolic activity. Yeast-derived components such as MOS may contribute to modulation of intestinal fermentation function, whereas specific preparations, such as iron-enriched *S. cerevisiae*, have been reported to improve iron utilization and redox homeostasis (151). In addition, *Wickerhamomyces* anomalus has been reported to facilitate the restoration of intestinal metabolic homeostasis and improve redox balance (121). Supplementation with yeast probiotics is typically associated with reduced diarrhea incidence, increased villus height, improved growth performance, and enhanced antioxidant capacity (152, 153). Sandrini et al. (154) demonstrated that a yeast blend improved ileal morphology and optimized the intestinal microenvironment, while Zeng et al. (155) showed that iron-enriched *S. cerevisiae* enhanced antioxidant capacity and promoted post-weaning recovery.

Notably, compared with single-strain preparations, some multi-strain probiotic formulations may exert complementary effects by targeting multiple microbial taxa and functional pathways; however, these effects are likely to depend on the specific strains and formulation (134, 156). Concurrently, probiotic consortia synergistically enhance key metabolic pathways, including SCFA production, bile acid metabolism, and tryptophan metabolism, facilitating the reconstruction of intestinal metabolic functions (60, 134). Improvements at the ecological and metabolic levels may contribute to better intestinal health, while specific probiotic strains or formulations have been associated with reduced diarrhea incidence, improved growth performance, and enhanced feed efficiency, although these production benefits may vary among strains and experimental conditions (157).

Emerging next-generation probiotics (NGPs), including *Akkermansia* and *Prevotella*, have recently attracted increasing attention because of their potential to reshape the gut microbial ecosystem and improve intestinal health (158). In weaning piglets, selected NGP candidates, such as *Akkermansia muciniphila* and *Prevotella copri*, have shown potential to alleviate diarrhea and improve intestinal function, highlighting their potential as alternative or complementary strategies to conventional probiotics (159, 160). However, compared with established probiotic genera, evidence regarding their strain-specific efficacy, safety, colonization characteristics, and practical application in weaning piglets remains limited. Therefore, NGPs represent a promising emerging direction rather than an established replacement for conventional probiotics.

Collectively, current evidence indicates that specific probiotic strains and formulations have substantial potential to alleviate weaning-associated intestinal dysfunction, with reported effects involving microbial ecological remodeling, changes in microbial metabolic functions, and improvements in host phenotypes (8, 26, 60, 133, 157). This integrated framework highlights the potential of probiotics as sustainable alternatives to AGPs in modern antibiotic-free swine production systems. Importantly, this proposed sequence should not be interpreted as a universally established causal cascade. While experimental studies have demonstrated causal roles for specific microbial metabolites or host signaling pathways in selected contexts, many reported associations between microbial composition, metabolic profiles, and host phenotypes remain mechanistically unresolved.

## 6 Key determinants and challenges affecting the efficacy of probiotic interventions

Although substantial evidence has demonstrated the potential of probiotics to alleviate weaning stress in piglets, their efficacy under practical production conditions remains highly variable across experimental settings and commercial production systems. This variability is influenced by host genetic background, dietary composition, and the intestinal microenvironment, which can shape probiotic survival, microbial interactions, metabolic activity, and host responsiveness.

### 6.1 Host breed differences

The host genetic background shapes the initial composition of the intestinal microbiota and influences probiotic colonization, persistence, and functional efficacy. Previous studies have shown that pigs with different genetic backgrounds exhibit marked differences in microbial diversity, dominant bacterial taxa, and microbial succession patterns. Comparative analyses have revealed that, relative to indigenous Chinese breeds, such as Lantang, Meishan, Erhualian, and Shanxi Black pigs, commercial breeds including Duroc, Landrace, and Large White pigs generally possess lower microbial diversity and less stable gut microbial ecosystems (161, 162). For instance, local breeds such as Meishan pigs often display greater resilience of intestinal barrier function during weaning stress, whereas Duroc and Large White pigs are more prone to developing pro-inflammatory immune responses (163). These host-associated microbial characteristics further influence the survival, persistence, and functional activity of exogenous probiotics. At the mechanistic level, host genetic variation may influence probiotic-microbiota interactions by shaping epithelial barrier properties, mucin production, innate immune responsiveness, and the availability of microbial niches. These host-dependent factors can alter colonization resistance and determine whether administered probiotics can establish functional interactions with resident microbial communities. Thus, breed-associated differences in the host-microbiota interface may contribute to variation in probiotic persistence and functional activity (164). Indigenous pig breeds typically harbor more complex and stable commensal microbial communities, resulting in stronger colonization resistance that may limit probiotic establishment. In contrast, the relatively less complex microbiota of commercial breeds may provide greater ecological opportunities for probiotic establishment or activity (162, 165). These observations suggest that host genetic background should be considered when designing precision probiotic supplementation strategies for commercial pig production.

### 6.2 Dietary composition

Dietary components not only serve as nutritional substrates for piglets but also strongly influence probiotic survival, persistence, metabolic activity, and interactions with the resident microbiota. Variations in diet composition alter substrate availability, luminal pH, and microbial metabolic activity, thereby influencing probiotic survival, cross-feeding interactions, and competition with resident microbes. These diet-dependent changes in the intestinal metabolic environment can consequently determine the functional activity and efficacy of administered probiotics.

Among dietary factors, fiber, particularly NSPs, plays a pivotal role in maintaining intestinal microbial homeostasis. NSPs serve as fermentable substrates that support cross-feeding interactions between probiotics and SCFA-producing commensal bacteria, thereby enhancing SCFA production. Through metabolic cross-feeding, these fiber components promote the growth of probiotics and other beneficial commensal microorganisms (166). In addition, an adequate supply of fermentable fiber helps meet the energy demands of the gut microbiota, preventing microbes from utilizing host-derived mucins as alternative energy sources. This contributes to the preservation of the mucus barrier and provides stable attachment sites for probiotic colonization (167, 168).

Conversely, excessive dietary protein levels in weaned piglets frequently compromise the efficacy of probiotic supplementation. Owing to insufficient digestive enzyme secretion during the early post-weaning period, substantial amounts of undigested protein reach the large intestine, where microbial putrefactive fermentation generates potentially harmful metabolites, including ammonia and hydrogen sulfide (169). These metabolites changes can alter the intestinal luminal environment and may favor the proliferation of opportunistic pathogens such as ETEC. Consequently, intensified competition for ecological niches and nutrient resources inhibits probiotic colonization and ultimately diminishes their protective effects on intestinal health (170, 171). Therefore, optimizing dietary composition, particularly through appropriate management of crude protein and fermentable fiber, may improve the intestinal microenvironment, enhance probiotic colonization efficiency, and consequently increase the effectiveness of probiotic interventions in mitigating weaning stress and post-weaning diarrhea.

### 6.3 Intestinal microenvironment and mitigation strategies

The survival, persistence, and functional activity of exogenous probiotics within the piglet intestine are strongly influenced by the pre-existing microbial ecosystem of the host. Indigenous gut microbiota can exert colonization resistance against incoming probiotic strains through niche occupation, competition for nutrients, and the production of antimicrobial compounds, thereby contributing to inter-individual and inter-population variability in probiotic efficacy (172). At the molecular level, luminal pH, bile acids, oxygen availability, redox status, mucus composition, and host-derived antimicrobial factors can influence probiotic membrane stability, metabolic activity, stress responses, and interactions with resident microbes (71, 173). These environmental constraints may therefore affect not only probiotic survival during gastrointestinal transit but also the functional activity of surviving cells within the intestinal microbial ecosystem (173, 174).

Furthermore, before reaching their target intestinal sites, probiotic microorganisms must withstand the highly acidic gastric environment, where pH values may fall to 2-3, as well as the elevated concentrations of bile salts present in the duodenum (175, 176). To overcome these challenges, recent research has focused on engineering advanced probiotic delivery systems. Lipid membrane encapsulation can modify the physicochemical properties of probiotic cell surfaces, enhancing their interactions with the intestinal mucosa and improving survival and retention within the mucus layer (177, 178). Likewise, microencapsulation and nanocoating technologies provide physical protection against gastric acid and bile salt-induced damage (179, 180). More importantly, these delivery platforms enable controlled release of probiotics at specific intestinal locations, thereby facilitating more precise targeting of suitable ecological niches. Such strategies can improve probiotic viability during gastrointestinal transit and may enhance their functional activity within the complex intestinal microenvironment (181). Collectively, these advances in delivery technology may substantially improve probiotic stability, colonization efficiency, and reproducibility under commercial production conditions. Overall, host genotype, dietary substrates, and intestinal physicochemical conditions interact to shape the ecological niches, metabolic resources, and colonization resistance that determine probiotic–microbiota interactions and ultimately influence probiotic efficacy.

## 7 Conclusions and future perspectives

Weaning stress represents a major challenge in pig production and is frequently associated with intestinal microbiota dysbiosis, impaired barrier function, and immune dysfunction in piglets, ultimately leading to post-weaning diarrhea, growth retardation, and reduced production performance. In the context of restrictions on in-feed antibiotic use, probiotics have emerged as a promising and sustainable alternative strategy. Collectively, current evidence demonstrates that probiotics restore intestinal homeostasis primarily through remodeling the gut microbiota and re-establishing microbiota-derived metabolic signaling. Furthermore, we highlight how probiotics alleviate weaning-associated stress through multiple complementary pathways, including enhancement of intestinal barrier integrity, maintenance of immune homeostasis, reinforcement of antioxidant defenses, and potential modulation of the gut-brain axis.

Despite these advances, several challenges continue to limit the widespread application of probiotics. Their efficacy remains variable, the underlying mechanisms are not yet fully understood, and substantial inter-individual differences in treatment responses persist. Most current studies remain largely descriptive, focusing on phenotypic outcomes, rather than establishing causal relationships between microbial alterations, metabolite production, and host signaling pathways, hindering the development of precision intervention strategies. In addition, probiotic safety requires systematic evaluation at the strain level before practical application. Safety assessment should include whole-genome screening for acquired antimicrobial resistance determinants, virulence-associated genes, and other potentially undesirable genetic traits, together with phenotypic characterization of antimicrobial susceptibility and relevant virulence properties. Particular attention should also be paid to the genomic context and mobility of antimicrobial resistance determinants, as transferable resistance genes may potentially be disseminated through horizontal gene transfer within the gut microbial community. Therefore, the absence of clinically relevant resistance determinants and virulence-associated traits, together with an appropriate safety profile, should be considered essential criteria for the selection and development of probiotic strains.

Future research should focus on several key directions. First, probiotic supplementation strategies may evolve from conventional strain-centered approaches toward microbiome-guided precision nutrition, in which probiotic selection is tailored according to individual microbial configurations and metabolic deficiencies. Second, integrated multi-omics analyses, including metagenomics, metabolomics, and host transcriptomics, should be employed to systematically elucidate the functional pathways and molecular targets underlying probiotic action. Third, the development of NGPs, including emerging commensal candidates such as *Akkermansia*, *Prevotella*, and butyrate-producing bacteria such as *Faecalibacterium prausnitzii*, warrants further investigation in weaning piglets. Translating these emerging approaches into practical swine production remains challenging. Key barriers include strain stability, gastrointestinal survival and persistence, biosafety, large-scale production, formulation and storage stability, regulatory requirements, cost-effectiveness, and consistency under commercial production conditions. Addressing these challenges will require standardized efficacy and safety evaluation, optimized formulation and delivery systems, and validation in large-scale field trials. In addition, combination strategies involving probiotics and complementary interventions, including prebiotics, postbiotics, and phytobiotics, may represent an effective and practical approach for the prevention and management of weaning stress in piglets. Ultimately, successful translation will depend on integrating microbiome-based approaches with scalable production, robust safety evaluation, and field validation to support the practical application of probiotics in sustainable swine production.
